# A Study to Assess Awareness of Physical Medicine and Rehabilitation Services Among the General Public

**DOI:** 10.7759/cureus.90855

**Published:** 2025-08-24

**Authors:** Abhishek Chowdhery, Hitendra Gupta, Aahan Singh Rana

**Affiliations:** 1 Sports Medicine, King George's Medical University, Lucknow, IND; 2 Physiology, Pandit Deendayal Upadhyay Government Medical College, Rajkot, IND; 3 Physical Medicine and Rehabilitation, Government Doon Medical College, Dehradun, IND

**Keywords:** education and communication, management, p.m.r awareness, p.m.r information, p.m.r (physical medicine & rehabilitation)

## Abstract

Aim: Rehabilitation medicine, or physical medicine and rehabilitation (PMR), constitutes an essential component of healthcare and is often recognized as the fourth pillar of the healthcare system. However, in India, public awareness of this medical specialty and its wide range of services remains limited. The Government of Uttarakhand, particularly the Government Doon Medical College and its associated hospital, has been making dedicated efforts to improve public awareness of the PMR department and its services. However, due to the absence of baseline data, there is a lack of clarity on how best to approach this challenge. Therefore, Government Doon Medical College and its associated hospital conducted this study to assess the current level of awareness and identify strategies to enhance it.

Objective: To assess the awareness and understanding of the physical medicine and rehabilitation (PMR) specialty, its scope, and available services among general patients and public visitors to Government Doon Medical College and its associated Doon Hospital. To identify participants' knowledge regarding the location, facilities, and specific interventions provided by the PMR department. To compare awareness levels across demographic subgroups (e.g., age, gender, education level). To evaluate the role of different information channels (e.g., hospital staff, media, community outreach) in creating awareness about PMR services.

Materials and methods: This observational study was conducted in the department of PMR at a multispecialty hospital in Dehradun, Uttarakhand. A panel of experts developed a 20-item questionnaire designed to assess awareness of PMR services and identify measures to improve public knowledge and referral practices. The questionnaire addressed general awareness of the specialty, its benefits, and the existing information, education, and communication (IEC) tools within the hospital.

Results: The findings revealed that the overall awareness of PMR services among participants was low (less than 50%). Additionally, the use of IEC tools within the hospital to disseminate information about PMR was also limited (utilization below 50%).

## Introduction

According to the World Health Organization (WHO), rehabilitation involves utilizing various methods to minimize the effects of disabling and handicapping conditions and to promote optimal social inclusion for individuals with disabilities [1,2]. Physical and rehabilitation medicine (PRM), also known as physical medicine & rehabilitation (PMR), is a medical specialty that places rehabilitation at the core of its healthcare approach [3].

PMR, also referred to as physiatry, focuses on the prevention, diagnosis, and treatment of disabling diseases, disorders, and injuries. In our country, there is a significant lack of information and awareness regarding rehabilitation medicine, leading to the underutilization of its services by both medical professionals and the general population [4].

PMR provides medical care for individuals who have experienced catastrophic injuries, undergone major surgeries, or are affected by diseases impacting the musculoskeletal or neurological systems, chronic pain, or limited mobility. Many countries, including ours, are witnessing a growing aging population, highlighting the essential role PMR can play in such societies [5,6]. The primary goal of PMR is to help individuals achieve optimal functional independence despite their limitations. Physiatrists lead multidisciplinary teams and develop strategies to maximize patients' recovery and functionality [7].

Physiatry helps patients regain the ability to manage daily life independently by restoring bodily function [8]. The PMR team includes not only physiatrists but also allied health professionals such as physiotherapists, occupational therapists, speech and language therapists, psychologists, orthotists, and prosthetists. The team's aim is not necessarily to restore full pre-illness or pre-injury function, but rather to enhance the patient's quality of life to the greatest extent possible.

In Dehradun, Uttarakhand, a growing and aging population has led to an increased incidence of disabilities-largely due to congenital disorders, chronic systemic diseases, and traumatic injuries such as those from road accidents. Consequently, the demand for PMR services is increasing, along with the need for greater public and professional awareness. Over the past two decades, the Ministry of Health (MOH) in Uttarakhand has initiated several rehabilitation programs within PMR departments to support individuals with disabilities and neuromusculoskeletal issues. However, many medical colleges across the state face challenges, including non-functional departments and insufficient staffing.

It is important to distinguish between PMR rehabilitation services and physiotherapy, as total rehabilitation requires a coordinated effort led by a PMR physician and supported by a multidisciplinary team.

PMR treatments typically include physical therapies, medications, and/or minimally invasive procedures. Improved functional recovery post-surgery has often been observed with PMR involvement [9]. Multiple studies have documented a growing need for more rehabilitation centers, trained rehabilitation physicians, and comprehensive rehabilitation teams. For example, in Saudi Arabia, the establishment of the Saudi PMR Board has significantly improved service effectiveness nationwide [10].

There is a noticeable lack of comprehensive research on public awareness of PMR in our country, particularly in Uttarakhand and more specifically in Dehradun. Therefore, the present study aims to assess the level of awareness among the general public and hospital patients regarding PMR at Government Doon Medical College and Doon Hospital in Dehradun, Uttarakhand, India.

## Materials and methods

Study area/setting

The study was conducted at Government Doon Medical College and its associated Doon Hospital.

Study design

This was a cross-sectional observational study.

Study period

The study was carried out from 1st November 2023 to 15th December 2023.

Study population

The target population comprised general patients and members of the public visiting Doon Hospital, focusing on their awareness and understanding of the physical medicine and rehabilitation (PMR) specialty, its scope, and available services. Inclusion of general patients and public visitors to the OPD ensures the target group for the primary objective.

Participants and data collection procedures

Participants were recruited from the outpatient department and public visitor areas of Doon Hospital, Dehradun, over a one-and-a-half-month period. Recruitment took place on weekdays between 9:00 AM and 4:00 PM at designated waiting areas. All individuals who met the inclusion criteria were approached in the order they arrived, until the daily target number was reached. Individuals who declined participation were thanked, and the reason for refusal (if voluntarily provided) was recorded anonymously to assess potential non-response bias. Trained research assistants approached eligible participants, explained the study purpose, and clarified that participation was voluntary with no impact on ongoing medical care. Those willing to participate received a participant information sheet and provided written informed consent. For participants unable to read or write, the form was read aloud in their preferred language (Hindi or English) and consent was obtained.

Study subjects

The study subjects were general patients and public visitors to Government Doon Medical College and its associated Doon Hospital. Demographic breakdowns and subgroup comparisons address secondary objectives relating to determinants of awareness.

Sample size

A total of 261 participants were included in this prospective study. Data were collected using a structured questionnaire administered to visitors between 1st November and 15th December 2023.

Sample size calculation 

The minimum sample size was calculated for a single estimate of proportions. This yielded n≈261n. We therefore included 261 participants. The finite population correction was not applied as the source population during the study period was large.

Sampling method

A consecutive sampling method was employed to recruit participants from the defined study population.

Study tool

A structured questionnaire was used to assess awareness of the PMR medical specialty, its scope, and services. The tool also evaluated the general level of awareness regarding PMR among participants. A structured questionnaire was adapted from previously published awareness surveys on physical medicine and rehabilitation (PMR) among healthcare providers, with modifications to suit the Indian healthcare context and institutional requirements. Content validity was established through review by a panel of three PMR specialists, one public health expert, and one biostatistician. The Content Validity Index (CVI) was calculated for each item, and questions with CVI < 0.80 were revised. A pilot test was conducted among 10 medical professionals (excluded from the final analysis) to assess clarity and relevance. Feedback was incorporated to refine wording and sequence. Internal consistency reliability was acceptable (Cronbach's alpha = 0.81).

Structured questionnaire items (see Appendix, Tables 4, 5) directly measure awareness, knowledge of services, and information sources for secondary objectives.

Inclusion criteria

General patients and public visitors to the outpatient department of Doon Hospital. Individuals who were not relatives of staff working in the Department of Physical Medicine and Rehabilitation.

Exclusion criteria

Individuals who were unwilling or did not provide consent to participate. Staff members or relatives of staff working in the Department of Physical Medicine and Rehabilitation.

Statistical analysis

Data were coded and entered into Microsoft Excel (Microsoft Corporation, Redmond, Washington, USA) for initial checks, followed by statistical analysis using IBM SPSS Statistics (IBM Corp., Armonk, New York, USA). Descriptive statistics were used to summarize demographic characteristics and awareness levels, while chi-square tests were employed to explore associations between categorical variables. A p-value of <0.05 was considered statistically significant.

## Results

A total of 261 respondents participated in the study. A comparative analysis of awareness of PMR services across demographic groups revealed statistically significant differences based on gender and educational qualification. Awareness was higher among males (38%) than females (28%) (p = 0.045) and significantly greater among postgraduates (55%) compared to undergraduates (40%) (p = 0.012). No statistically significant difference in awareness was observed across age groups (p = 0.31) (Table 1).

**Table 1:** Awareness of PMR services by demographic factors. PMR: physical medicine and rehabilitation.

Variable	Aware (%)	Not aware (%)	p-value
Gender			
Male	38%	62%	0.045*
Female	28%	72%	
Education			
Undergraduate	40%	60%	0.012*
Postgraduate	55%	45%	
Age group			
<30 years	30%	70%	0.31
>45 years	35%	65%	

The study included 261 participants, with the majority under 30 years of age (49%), and 60% being male. Most respondents were from Dehradun, Uttarakhand (71%) and held undergraduate (49%) or postgraduate (31%) qualifications. About half were unmarried (50%), with employment (34%) and student status (32%) being the most common occupations. Awareness of PMR services was generally low: while 56% had heard of the PMR specialty, only 34% were aware of the services, and just 38% knew the department's location. Awareness of PMR's distinction from physiotherapy was limited (46% were unaware), and only 26% were familiar with its latest services (Table 2).

**Table 2:** Respondent demographics and awareness of PMR services (n = 261). PMR: physical medicine and rehabilitation.

Variable	Category	N (%)
Age group	<30 years	128 (49%)
	>45 years	44 (17%)
Gender	Male	157 (60%)
	Female	104 (40%)
Location	From Dehradun, Uttarakhand	186 (71%)
Education	Undergraduate	128 (49%)
	Postgraduate	81 (31%)
Marital status	Unmarried	131 (50%)
	Married	123 (47%)
Occupation	Employed	89 (34%)
	Students	84 (32%)
Heard of PMR specialty	Yes	146 (56%)
	No	115 (44%)
Awareness of PMR department in hospital	Not aware	144 (55%)
Known location of PMR department	Yes	99 (38%)
Awareness of PMR services	Yes	89 (34%)
	Not sure	31 (12%)
	Not aware	68 (26%)
Aware of PMR vs physiotherapy difference	Not aware	120 (46%)
Aware of latest PMR services	Aware	68 (26%)
	Not aware	167 (64%)
Diseases treated in PMR	General awareness	139 (53%)
	Neuro	42 (16%)
	Musculoskeletal	50 (19%)
Awareness of rehab treatment modalities	General awareness	91 (35%)
	Limited to therapy	78 (30%)
Visited PMR department	No	151 (58%)
Seen PMR display boards in hospital	No	170 (65%)
Informed by other specialties about PMR	No	162 (62%)
Informed by paramedical/allied staff	No	151 (58%)
Referred to PMR by any department	No	170 (65%)
PMR info on hospital website	Not sure/not aware	205 (78%)
Informed by OPD staff	No	146 (56%)
Seen PMR info on social media	No	165 (63%)
Seen PMR booklet in hospital	No	180 (69%)
Seen PMR medical camp	No	170 (65%)
Referred any patient to PMR	No	136 (52%)

In terms of communication, the majority had not seen PMR displays (65%), been informed by other specialties (62%), or seen social media posts (63%) or hospital booklets (69%) related to PMR. Only 42% had any specific awareness of conditions treated within PMR (neuro or musculoskeletal), and 52% had never referred a patient to the department. These findings highlight a significant gap in patient and staff education regarding PMR services (Table 3).

**Table 3:** Statistical tests (χ², t, and F). PMR: physical medicine and rehabilitation.

Variable	Category	N (%)	Statistical test	Value
Gender vs PMR awareness	Male: 157 (60%), aware: 89	Chi-square	χ² = 4.23	0.04*
	Female: 104 (40%), aware: 57			
Education vs PMR awareness	UG: 128 (49%), aware: 70	Chi-square	χ² = 6.15	0.01*
	PG: 81 (31%), aware: 49			
Age (<30 vs >45) and awareness	<30: 128, aware: 61	Chi-square	χ² = 1.82	0.18
Employment vs PMR awareness	Employed: 89, aware: 40	Chi-square	χ² = 2.91	0.08
Awareness scores by gender	Awareness score (mean ± SD)	t-test	t = 2.07	0.04*
Awareness scores by age group	Mean ± SD (<30 vs >45)	ANOVA (F-test)	F = 3.44	0.036*
*Statistically significant at p < 0.05				

Statistical analysis revealed significant associations between certain demographic variables and awareness of PMR services. A chi-square test showed that gender was significantly associated with awareness, with males demonstrating higher awareness levels than females (χ² = 4.23, p = 0.045). Similarly, education level was a significant factor, with postgraduates exhibiting greater awareness compared to undergraduates (χ² = 6.15, p = 0.012). No significant differences were observed in awareness based on age group or employment status. However, independent t-test results indicated a statistically significant difference in mean awareness scores between genders (t = 2.07, p = 0.04), and ANOVA showed significant variation in awareness scores across age groups (F = 3.44, p = 0.036).

## Discussion

Physical medicine and rehabilitation (PMR) physicians, also known as physiatrists, manage a wide range of conditions affecting the brain, spinal cord, muscles, joints, and tendons. They are uniquely trained to lead multidisciplinary care teams aimed at enhancing patient functionality and quality of life [11]. Physiatrists design comprehensive treatment plans that include medical management, therapeutic exercise, assistive devices, and non-surgical interventions. These plans are implemented in collaboration with physical, occupational, and speech therapists, psychologists, vocational counselors, and rehabilitation nurses [12].

PMR is a distinct medical specialty headed by a medical specialist known as a physiatrist. Although this specialty has existed for quite some time, its integration and recognition across the country remain limited, often confined to select government institutions. Many still mistakenly equate rehabilitation services solely with physiotherapy. However, effective rehabilitation requires a collaborative team, which includes not only physiatrists but also allied health professionals such as occupational therapists, physiotherapists, speech therapists, clinical psychologists, vocational counselors, social workers, rehabilitation nurses, biomechanists, prosthetists, orthotists, and engineers.

In India, despite PMR being formally integrated into the medical curriculum, awareness of this specialty among healthcare professionals and the general public remains inconsistent. This lack of awareness contributes to underutilization of services and poor referral practices within hospital settings [13].

PMR encompasses medical, rehabilitative, and in some cases, surgical interventions aimed at enhancing quality of life. However, due to limited public knowledge and awareness, these services remain largely underutilized. Multiple factors contribute to this gap in awareness. This study aimed to establish a baseline understanding of public awareness regarding the PMR specialty. The findings reveal a significant lack of awareness, not only among the general public but also among hospital staff, including physicians, surgeons from other departments, allied health professionals, and administrative personnel.

A major finding was the widespread confusion between PMR as a medical specialty and physiotherapy as a standalone service. There was also limited knowledge of the broad range of services offered within PMR departments, which often leads to a lack of referrals from doctors and minimal self-referral by patients. Notably, multidisciplinary rehabilitation has consistently shown better outcomes compared to single-discipline approaches, reinforcing the importance of team-based, individualized care [14].

The inclusion of PMR in medical education represents a significant step toward making comprehensive rehabilitation care more widely accessible to the public [15].

Theoretical perspective: Application of the Health Belief Model (HBM)

The results of this study can be interpreted through the Health Belief Model (HBM), which explains health-related behaviors based on individuals' perceptions of susceptibility, severity, benefits, barriers, cues to action, and self-efficacy.

Perceived susceptibility and severity: Many individuals, including healthcare providers, may not perceive themselves or their patients as at risk of conditions that could benefit from PMR, or they may underestimate the severity of disability without timely rehabilitation.

Perceived benefits: Limited awareness of the scope and positive outcomes of PMR services diminishes the perceived value of seeking or recommending these services.

Perceived barriers: Misidentifying PMR as physiotherapy, lack of departmental visibility, and minimal curricular exposure act as barriers to utilization.

Cues to action: Hospital signage, physician endorsements, medical curriculum inclusion, awareness camps, and targeted media campaigns can serve as important triggers for both public and professional engagement.

Self-efficacy: Confidence in referring patients or seeking PMR services is likely to increase when individuals understand the department's capabilities and processes.

By framing PMR awareness initiatives within the HBM, interventions can be strategically targeted to modify perceptions, reduce barriers, and enhance both patient self-referral and professional referral practices.

Limitations

This study faced several limitations. The sample size was relatively small, limiting the generalizability of the findings. Additionally, because participants were selected from a specific area within the hospital, selection bias may have affected the results. Broader, multicenter surveys are necessary to obtain more representative and reliable data on PMR awareness. Another limitation was the absence of follow-up with participants who reported negative or limited knowledge about PMR. Understanding the reasons behind their responses could help improve interprofessional collaboration and awareness of PMR services across medical specialties.

Furthermore, the study lacks a comprehensive conceptual or theoretical framework to explain why awareness is low or how it might be improved. While the HBM was applied retrospectively to interpret results, future research should incorporate such models prospectively in study design. Additionally, there was minimal engagement with existing literature on health communication, behavior change, or medical education, which could have provided deeper insights into intervention strategies.

## Conclusions

Every crisis presents an opportunity, and in the context of physical medicine and rehabilitation (PMR), there is a pressing need to strengthen its presence within the medical undergraduate curriculum. Doing so will not only improve referral patterns among physicians but also enhance general public awareness of the specialty. We recommend that similar awareness surveys be conducted across various regions of the country to build a robust national dataset. This would enable a more comprehensive understanding of the current awareness levels and help guide strategic planning in PMR service development and integration.

To further promote PMR, the following initiatives are suggested: Expand PMR residency programs to meet the growing demand for trained physiatrists. Increase public engagement through medical camps offering PMR services, enhanced visibility on hospital websites, and active social media outreach. Foster interdisciplinary collaboration by organizing short-term PMR rotations for other medical professionals, participating in national conferences, and conducting continuing medical education (CME) sessions. Enhance visibility within hospitals through the display of PMR services, patient testimonials, newsletters, and peer-sharing of patient experiences within communities. Rehabilitation is truly the need of the hour. However, for India to realize the full potential of PMR, it must modernize its medical education, revamp existing policies, and invest in both physician and public awareness. This study aims to contribute toward these goals by shedding light on the current gaps and offering a path forward for improving PMR services and visibility across the healthcare system.

## Appendices

**Table 4:** Specialty awareness questionnaire. PMR: physical medicine and rehabilitation; EMG: electromyography; NCV: nerve conduction studies; CNS: central nervous system.

Question	Responses
1. What is your highest medical qualification?	Medical graduate
	Postgraduate
	Fellow
2. What is the department/specialty where you work?	Clinical
	Paraclinical
3. Have you heard about PMR as a medical postgraduate specialty?	Yes
	No
4. Do you have a department of PMR in your hospital?	Yes
	No
5. What do we call a medical doctor with MD in PMR?	Medical doctor
	Physiatrist
	Physiotherapist
	Do not know
6. What is the source of your knowledge about PMR as a medical specialty?	Medical school
	Residency training
	Medical studies
	Medical conferences
	Mass media, self-training, others
	Colleagues
7. Are you aware of the following diagnostic/invasive/advanced interventions being provided by a PMR doctor?	Skull traction
	Peri-articular and intra-articular injections
	Nerve blocks
	Botulinum toxin injections
	Platelet-rich plasma injections
	Ultrasound/image-guided injections
	EMG, NCV and other electrodiagnostic tests
	Musculoskeletal ultrasound
	Urodynamic evaluations
	Instrumental gait analysis
	Corrective surgical procedures for
	musculoskeletal problems
	Cystoscopy in neurogenic bladder
	Neuroprosthetic implants
	None of the above
8. Have you ever referred your patient to a PMR doctor?	Yes
	No
9. What are your reasons for referral to a PMR doctor?	Therapeutic exercises
	Physical modalities
	Electrodiagnostic studies
	Orthosis/prosthesis prescription
	Pain management
	Rehabilitation of CNS disorders
	Diagnosis and rehabilitation of myopathies–
	neuropathies
	Prevention, diagnosis and treatment of musculoskeletal and rheumatic disorders
	Geriatric rehabilitation
	Cardiopulmonary rehabilitation
	Never referred
10. Do you believe that the late inclusion of PMR in MBBS curriculum would help in changing the overall perception of healthcare professionals towards PMR?	Yes
	No
	Cannot say

**Table 5:** General awareness. PMR: physical medicine and rehabilitation.

Sl. No	Component	Response
1	Name	
2	Age	<30 years/30-45 years/>45 years
3	Gender	Male/Female
4	Residence	
5	Educational qualification	No formal education/Primary/Secondary/Undergraduate/Postgraduate
6	Marital status	Married/Divorced/Widowed/Single
7	Occupation	Self-Business/Homemaker/Employed/Student/Unemployed
8	Are you aware of a medical specialty PMR (physical medicine and rehabilitation)	Yes/No
9	Are you aware of the existence of department of PMR in your hospital?	Yes/No
10	Are you aware of exact location of PMR department in your hospital premises?	Yes/No
11	Are you aware of the facilities/services provided within the department of PMR	Yes/No/Not sure/To some extent
12	Is PMR services and physiotherapy services same or different	Same/Different
13	Are you aware of the latest services available within department of PMR (Artificial limb center, Musculoskeletal USG Diagnostic/therapeutic procedure, Gait lab, Virtual reality and others)?	Yes/No/To some extent/Not sure
14	Does PMR Department carry out any surgery	Yes/No
15	What type of disease entities PMR department looks into	Musculoskeletal/Disability related issues/Neurological/Surgeries/All of the above
16	What do you understand by rehabilitation treatment	Medicine treatment/Surgical treatment/Therapy/Allied health therapist treatment/All of the above
17	Have you ever visited the department personally	Yes/No
18	Have you come across any display/standee describing about the services of PMR department in hospital premises	Yes/No
19	Have you heard from any other department physician/surgeons for the services available in PMR	Yes/No
20	Have you heard from any other paramedical/allied health science staff about the services available in PMR?	Yes/No
21	Have you ever been referred to PMR department by other department	Yes/No
22	Does hospital website give information of PMR services	Yes/No/Not Sure
23	Have OPD counter staff have informed you about PMR services	Yes/No
24	Have you come across the social media post related to PMR services	Yes/No
25	Have you come across educational booklet on PMR available in hospital	Yes/No
26	Have you come across any camp organized in your community have the services of PMR	Yes/No
27	Have you ever recommended any patient for PMR services	Yes/No
